# Review of surveillance systems for tephritid fruit fly threats in Australia, New Zealand, and the United States

**DOI:** 10.1093/jee/toad228

**Published:** 2023-12-23

**Authors:** John M Kean, Nicholas C Manoukis, Bernie C Dominiak

**Affiliations:** Ruakura Science Centre, AgResearch, 10 Bisley Rd, Hamilton 3214, New Zealand; Daniel K. Inouye U.S. Pacific Basin Agricultural Research Center, Agricultural Research Service, United States Department of Agriculture, 64 Nowelo St, Hilo 96720, HI, USA; New South Wales Department of Primary Industries, The Ian Armstrong Building, 105 Prince St, Orange 2800, Australia

**Keywords:** biosecurity, lures, parapheromones, toxicants, trapping

## Abstract

Many countries conduct fruit fly surveillance but, while there are guidelines, practices vary widely. This review of some countries in the Pacific region demonstrates the diversity of fruit fly surveillance practices. All utilize 3 parapheromones—trimedlure, cuelure, and methyl eugenol—to trap adult male fruit flies. Some target species are not attracted to these compounds so other attractants such as food-based lures are used in certain areas or circumstances. Lure loading and replacement cycles depend on the target species and the local climate. Malathion and dichlorvos (DDVP) are commonly used toxicants, but not in all countries, and other toxicants are being developed to replace these older-generation pesticides. Jackson and Lynfield are commonly used trap designs but newer designs such as cone and Biotrap are being adopted. Local factors such as chemical registrations and climate affect the choice of trap, lure, dispenser, toxicant, and bait concentration. These choices affect the efficacy of traps, in turn influencing optimal trap deployment in space and time. Most states now follow similar practices around trap inspection, servicing, and data handling, but these processes will be disrupted by emerging automated trap technologies. Ultimately, different practices can be attributed to the unique fruit fly risk profiles faced by each state, particularly the suite of fruit flies already present and those that threaten from nearby. Despite the diversity of approaches, international trade in fruit continues with the assurance that fruit fly surveillance practices evolve and improve according to each country’s risk profile and incursion experience.

## Introduction

As the world tries to feed the burgeoning human population, trade and travel have resulted in accelerating international spread of insect pests (Venette and Hutchison 2021). Some of the most critical invasive insects are the fruit flies (Diptera: Tephritidae) which cause direct economic impact on a wide range of fresh horticultural commodities (e.g., fruit and vegetables, hereafter “fruit”) (Follett et al. 2021). Many countries impose stringent quarantine restrictions to prevent their entry (Follett and Neven 2006). Therefore, the detection of an alien fruit fly can disrupt domestic and international trade and usually triggers a regulatory response to prevent establishment or eradicate an incipient population (Suckling et al. 2016).

Fruit fly surveillance is conducted in most countries in the Pacific region, and elsewhere in the world. Here, we compare and contrast the operational details of the fruit fly surveillance systems of 4 Pacific countries/states (hereafter “states”)—Australia, California, Hawaii, and New Zealand (NZ)—and briefly touch on aspects from other jurisdictions. The origins and nature of fruit fly risks differ markedly between the 4 states. While Hawaii manages several well-established exotic species, NZ is free of all economically important fruit flies and focuses on early detection and eradication of occasional incursions. California experiences relatively high invasion pressure, and Australia’s states vary with several endemic pests in the north and fruit fly freedom in the south. All 4 states conduct surveillance programs based on identifying, assessing, and ranking the flies of greatest threat. The target species determine which lure types are used. Once attracted to the vicinity of the traps, trap architecture and toxicants are important to maximize fly capture. Trap placement and frequency of inspection may optimize early detection. However, investment in surveillance resources (traps, lures, labor, etc.) must be balanced against the level of threat and potential damages of an incursion.

**Fig. 1.**
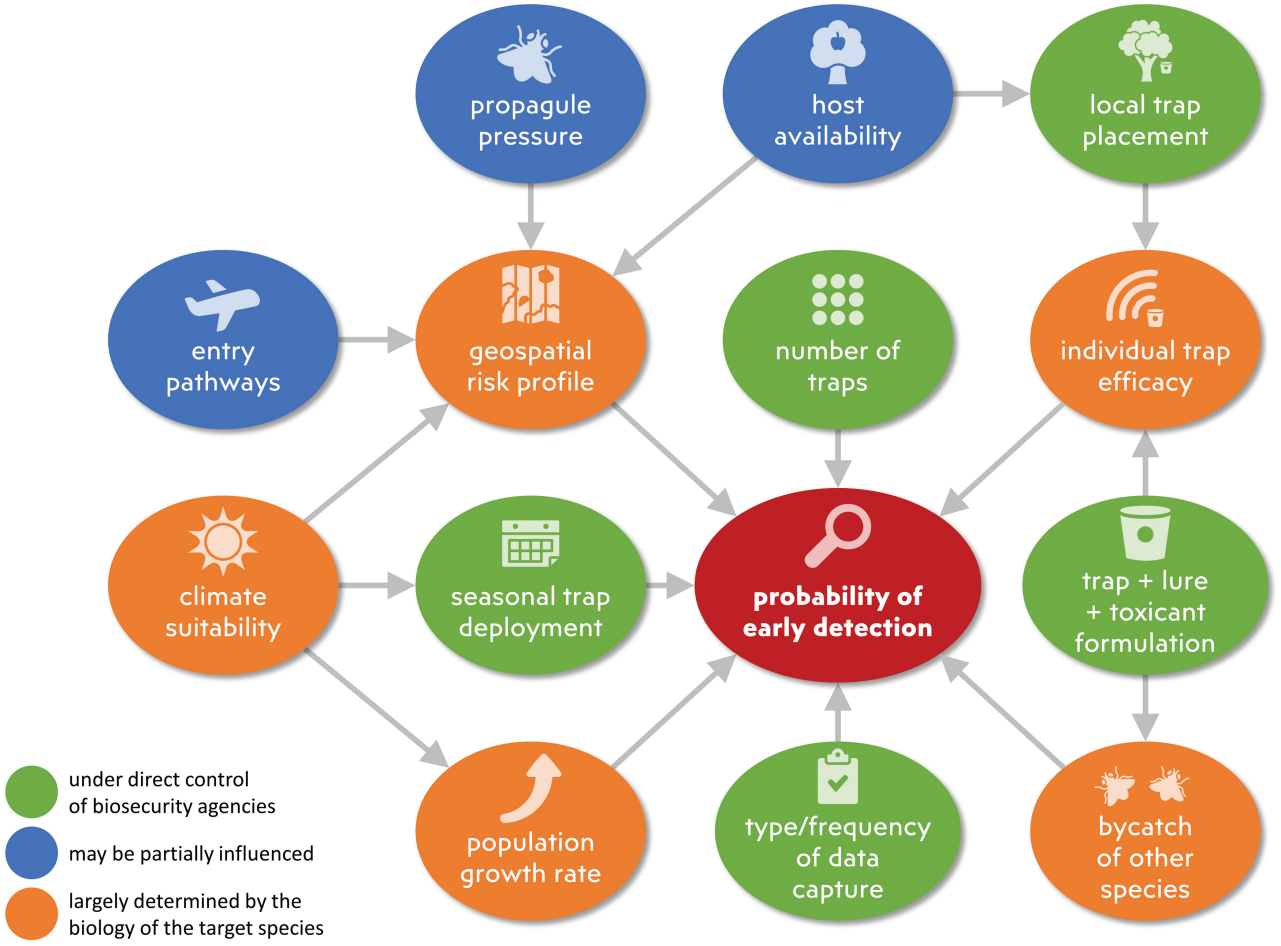
Main factors contributing to the probability of early detection of a fruit fly infestation.

Figure 1 summarizes some of the main factors contributing to the probability of detecting an incipient fruit fly population early. Our review is structured to assess many of these different factors and to demonstrate the similarities and differences among the approaches taken in 4 states with some of the most active biosecurity systems: NZ, Australia, Hawaii, and California. For these jurisdictions, we provide specific details to complement and update the global review of exotic fruit fly trapping networks by Quilici and Donner (2012), and synthesize recent research that is relevant to fruit fly surveillance. We show how current practices in each state continue to evolve to reflect previous experiences, perceived threats, and budget justification. These surveillance programs all achieve a level of assurance accepted by importing countries, despite differing in many details. The components in Fig. 1 demonstrate the complexity of fruit fly surveillance, and that there is no “one size fits all” approach to tephritid fruit fly surveillance.

## Target Species and Risk Assessment

### Threats to New Zealand

**Fig. 2.**
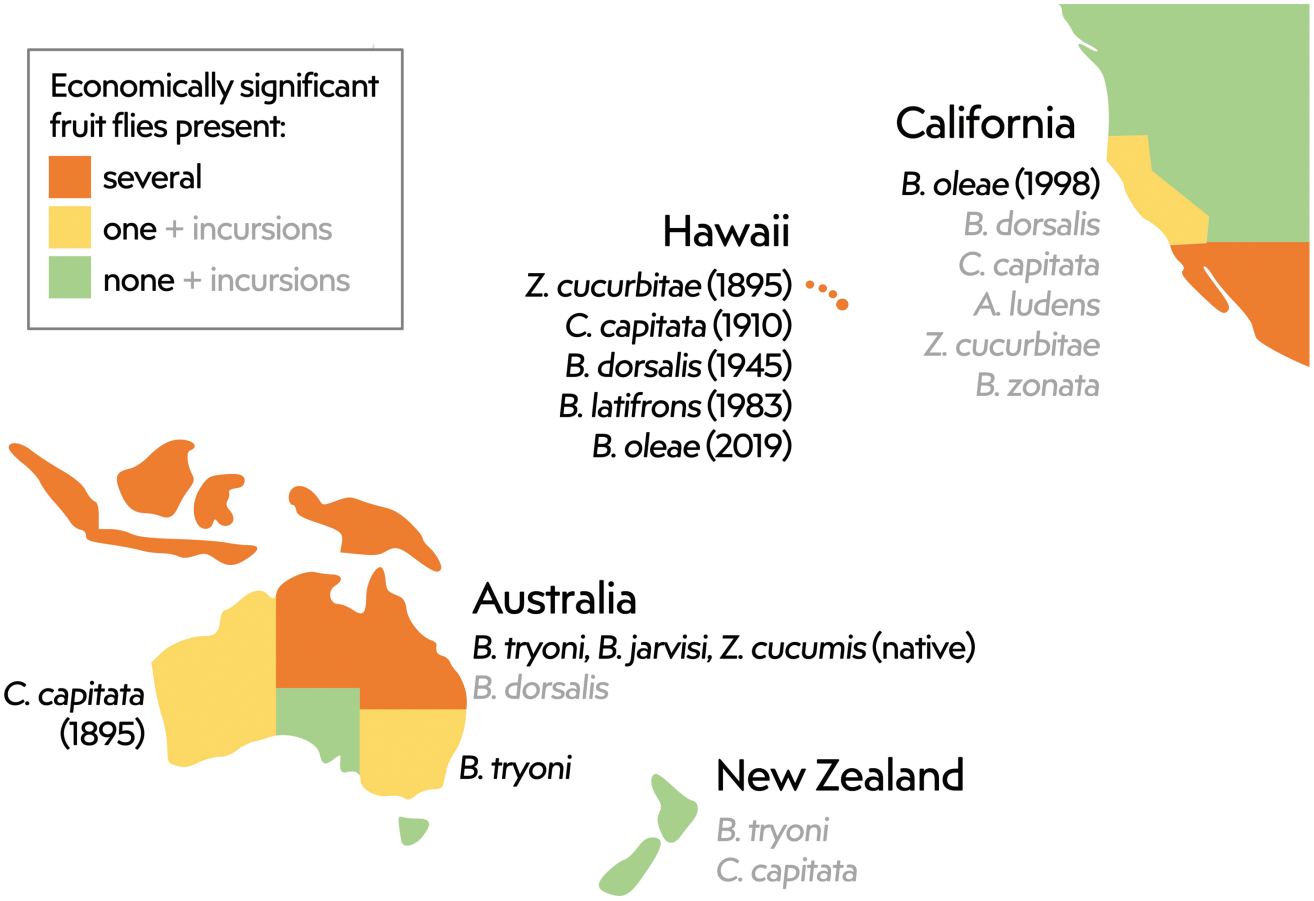
Summary of the main fruit flies of economic significance present in each of 4 Pacific states. Dates indicate when each species invaded and became established. Species listed in grey have a history of post-border detection, but authorities accept that these have failed to establish or have been eradicated.

Fruit flies are regarded as one of the most significant biosecurity threats to NZ’s horticultural industries, which have a combined export value of over NZD 4 billion per annum. Of our 4 chosen states, only NZ is free from fruit flies of economic importance (Fig. 2), and we focus frequently on NZ’s effective surveillance system for early detection.

There are multiple sources of fruit fly risk for NZ (Popa-Báez et al. 2021). Australia, NZ’s closest neighbor and a major trading partner, has c. 90 fruit fly species, about 10 of which are of economic concern. Of these, the most damaging is Queensland fruit fly, *Bactrocera tryoni* (Froggatt), which is present in non-arid eastern Australia (Dominiak and Mapson 2017). This species has been detected in NZ on 8 occasions, including an established population in Auckland in 2015 which was successfully eradicated (Kean 2016). Lesser Queensland fruit fly, *Bactrocera neohumeralis* (Hardy) has a similar host range and is also present along the eastern Australian coast north of Sydney (Dominiak and Worsley 2016). Jarvis fruit fly, *Bactrocera jarvisi* (Tryon), is endemic in Queensland and possibly north coastal New South Wales (NSW) (Dominiak and Worsley 2017). However, these latter 2 species have never been detected in NZ.

Mediterranean fruit fly (*Ceratitis capitata* Weidemann) established and was eradicated from at least 4 places in NZ in 1907 (New Zealand Department of Agriculture 1907, 1908), when it was widespread in southeastern Australia (Dominiak and Daniels 2012). This species is no longer present in eastern Australia, reducing the risk of introduction to NZ. Nevertheless, *C. capitata* was established in Auckland in 1996, with haplotyping and pathway analysis suggesting the origin of this incursion was Hawaii (George Gill, MPI, pers. comm.). This infestation was successfully eradicated (Holder et al. 1997). Together, *C. capitata* and *B. tryoni* comprise 11 of the 15 documented events of post-border detection of fruit flies in NZ since the early 20th century (MacLellan et al. 2021).

A lesser threat from Australia is cucumber fruit fly, *Zeugodacus cucumis* (French), which is established in Queensland down to the NSW border (Fay et al. 2022). New Zealand revised its Import Health Standards to mitigate the risk on commercial pathways from *Z. cucumis*, *B. latifrons* (Hendel), *B. tsuneonis* (Miyake), and *Anastrepha* spp. (Ministry of Agriculture and Forestry 2009).

There are many Asian fruit flies that pose a risk to NZ from other trading partners. Oriental fruit fly [*Bactrocera dorsalis* (Hendel)] is considered the greatest fruit fly threat to NZ and Australia from Asia, and (as its synonym *B. papayae*) was trapped in NZ in 1996. At that time, *B. dorsalis* had infested some 850,000 ha of Queensland, Australia (Cantrell et al. 2002), and this was the likely source of the NZ incursion. Since then, *B. dorsalis* has been eradicated from Australia (Cantrell et al. 2002, Suckling et al. 2016), reducing the risk of introduction to NZ. However, *B. dorsalis* remains of critical concern to both Australia and NZ due to its demonstrated invasion ability, competitiveness with other fruit flies, and wide host range (Duyck, Sterlin et al. 2004, Clarke et al. 2005, De Villiers et al. 2016).

The remaining NZ trap detections were tropical Pacific species: Fijian fruit fly *Bactrocera passiflorae* (Froggatt) in 1990, *Zeugodacus tau* (Walker) in 2016, and *B. fascialis* in 2019 (MacLellan et al. 2021). The first of these is widespread in Fiji and present also in Niue, Tonga, Tuvalu, and Wallis & Futuna Islands, the second is widespread in eastern Asia, and the third is known only from Tonga (Clarke et al. 2004).

### Threats to Australia

For Australia as a whole, the main exotic threat comes from South-East Asia because NZ is free of damaging fruit flies. Therefore, Australian surveillance is conducted primarily for domestic and exotic Asian tephritids. In October 1995, the exotic *B. papayae* (Drew and Hancock) was detected near Cairns in northern Queensland (Gillespie 2003). Shortly afterward in November 1997, *B. philippinensis* (Drew and Hancock) was detected in Northern Territory. Both incursions were eradicated (Hancock 2013). These 2 species were later synonymized into *B. dorsalis* (Schutze et al. 2015) and a possible future incursion of *B. dorsalis* is the primary driver for continued surveillance in all first ports of entry (Dominiak 2020).

Within Australia, fruit fly threats vary from state to state. *Bactrocera tryoni* is endemic along the eastern Australian coast (Dominiak and Mapson 2017). Producers wishing to sell produce in areas free from *B. tryoni* (domestically or internationally) must demonstrate that their property is free from *B. tryoni*. One advantage of *B. tryoni* being endemic is that it appears to exclude *C. capitata* from establishing (Dominiak and Mapson 2017). Southeastern Australia is concerned that “northern” flies such as *B. jarvisi* and *Z. cucumis* may establish south of the Queensland border, and use traps to monitor any range changes, possibly due to climate change (Sultana et al. 2017, 2020, Dominiak 2020, Simpson et al. 2020).

Northern Queensland fruit flies, such as mango fruit fly *Bactrocera frauenfeldi* (Schiner) (Royer et al. 2016) and *B. aquilonis* (May), have not been detected in NSW or NZ and are unlikely to pose a threat to these areas under current climates. Cucumber fruit fly, *Z. cucumis*, is a hot climate pest and until recently has not persisted south of Queensland’s boundary with NSW (Fay et al. 2022).

### Threats to Hawaii

Hawaii has long been severely impacted by Tephritids of economic importance, starting with the introduction of melon fly (*Zeugodacus cucurbitae* Coquillett) around 1895, followed by *C. capitata* in 1910. These 2 species had a significant impact on the diversified agriculture of the islands, as evidenced by the establishment of a “fruit fly investigations” laboratory in Honolulu by the United States Department of Agriculture in the first decade of the 20th century. *Bactrocera dorsalis* was accidentally introduced in 1945 and has remained one of the most serious fruit pests in the state. Additional responses included extensive efforts in biological control, with an unprecedented 32 braconid wasp species introduced to Hawaii between 1947 and 1952 specifically targeting invasive tephtritids (Vargas, Leblanc, et al. 2012). Still, incursions and establishments of fruit flies have continued. Solanaceous or Malaysian fruit fly (*Bactrocera latifrons* [Hendel]) was detected in 1983. More recently, olive fruit fly (*Bactrocera oleae* [Rossi]) was detected for the first time on Hawai’i and Maui islands in 2019 (Matsunaga et al. 2019). Internal quarantines against many of these pests slowed but did not halt their spread, and, except for the most recent arrival, these species have now infested all the major Hawaiian Islands. Hawaii implemented a successful area-wide pest management program against these pests (Vargas et al. 2008), but this could be threatened by further fruit fly incursions.

Hawaii has a very high volume of visitors, peaking at over 10 M in 2019 (Hawaii Tourism Authority 2022). Since air travel is a significant pathway for fruit fly invasion (Liebhold et al. 2006), Hawaii poses a significant threat of fruit fly spread to the mainland USA and other areas. Hawaii is thought to be the source of the *C. capitata* incursion in NZ in the 1990s.

Surveillance via trapping for additional species that threaten Hawaii, such as *Zeugodacus tau* (Enderlein) or *Bactrocera zonata* (Saunders) (species that respond to the male lures cuelure and methyl eugenol, respectively), is complicated by the fact that there are already established populations of cuelure and methyl eugenol responders (as well as Mediterranean fruit fly, which respond to trimedlure). Due to high population sizes, traps baited with male lures are quickly overwhelmed with hundreds or thousands of individuals of the established species.

### Threats to California

California has *B. oleae* established (Rice et al. 2003), but the permanent presence of breeding populations of other species of economic importance is not generally accepted. There are regular detections of multiple species of fruit flies in California, including *B. dorsalis*, *C. capitata*, Mexican fruit fly (*Anastrepha ludens* Loew), *Z. cucurbitae*, and peach fruit fly (*B. zonata* [Saunders]) (Fig. 2). This has led some authors to suggest that some of these species may be widely established in California at sub-detectable levels (e.g., Papadopoulos et al. 2013). However, most researchers and authorities consider this to indicate frequent incursions that fail to persist due to trapping and response (Mcinnis et al. 2017). Clearly, propagule pressure is very high (Liebhold et al. 2006), and California’s border biosecurity measures for fruit flies are less stringent than those of Australia and NZ.

California is the major producer of fresh fruit in the United States of America, and the establishment of a species such as *C. capitata* or *B. dorsalis* would cause billions of dollars of economic damage in the first years alone (Suckling et al. 2016). For this reason, the state maintains a robust surveillance network based on adult trapping to guard against spread following incursions. Trapping and detection procedures are comprehensively detailed by the California Department of Food and Agriculture (Gilbert et al. 2013), and the state operates a network of approximately 95,000 traps against tephritids (Quilici and Donner 2012). Since the mid-1990s, California also has operated a preventative release program of sterile *C. capitata* throughout the greater Los Angeles area to help reduce the number of incursions that established (Barry et al. 2004).

### Changing Risk Factors

Changes in international distributions of fruit fly species, together with shifting import and tourism patterns mean that the risk profiles of particular fruit fly species are constantly changing. For example, *C. capitata* was a very common interception in Australian fruit imports to NZ a century ago (New Zealand Department of Agriculture 1907) but is now confined to Western Australia (Dominiak and Daniels 2012) and poses a lower risk of introduction to NZ. Similarly, the single oriental fruit fly (as *B. papayae*) trapped in Auckland in 1996 likely originated from the large infestation in Queensland which was later eradicated, so oriental fruit fly is no longer a threat from this region. Conversely, the geographic range of *B. tryoni* has increased in recent years, arguably due at least in part to climate change (Dominiak and Mapson 2017, Simpson et al. 2020). There is some indication that *Bactrocera bryoniae* (Tryon) has extended its range in 2020/2021 to become established in Sydney (Dominiak and Millynn 2022). Another factor that can change risk calculations is shifts in dominant fruit fly species in source regions due to subsequent establishments. Complex shifts in abundance and frequency of established exotic fruit fly pests have been observed in La Réunion island with subsequent introductions of other species (Charlery De La Masselière et al. 2017). The dependence of introduction risk on fruit fly status of nearby and trading partners (Deschepper et al. 2021) highlights the need for states to work together to mitigate fruit fly threats.

Local climate at the destination of incursions is another key component of risk (Stephens et al. 2016), including the effects of artificial irrigation (e.g., Szyniszewska et al. 2020). In Australia, modeling has identified that the optimal climatic niche for *B. tryoni* is moving south, primarily along the coast (Sultana et al. 2017, Simpson et al. 2020). Parts of Victoria are predicted to become increasingly suitable for *B. tryoni* and productive tablelands to become increasingly susceptible to *B. tryoni* establishment. As winters become warmer, more *B. tryoni* adults are predicted to survive winter, and the flight threshold and successful matings are likely to occur earlier in each spring. A lengthened and warmer season may result in more generations per year and increased population sizes (Simpson et al. 2020). Also, shifts in rainfall patterns will affect *B. tryoni* populations. The 2010/2011 season was the wettest 2-year period in NSW’s history and resulted in widespread *B. tryoni* outbreaks in the Victoria and NSW Fruit Fly Exclusion Zone (FFEZ). Despite considerable expenditure of funds and resources, it became clear that fruit fly freedom was no longer technically feasible or economically sustainable there, and the NSW portion of the FFEZ was closed in 2013 (Dominiak and Mapson 2017). Similarly, NZ and Tasmania can no longer rely on past environmental conditions to limit fruit fly establishment nor freedom in the case of incursions. However, now that *B. tryoni* is endemic through all of eastern Australia, there may be relatively little further increase in propagule pressure on NZ from its range increase.

Climate may play an additional significant role in mediating competitive interactions between invasive fruit flies. When *B. dorsalis* invaded Hawaii, it displaced *C. capitata* throughout much of its range (Duyck, David, et al. 2004, Vargas et al. 2016), especially at lower elevations. However, *C. capitata* remained dominant in areas without high numbers of *B. dorsalis* hosts (Vargas et al. 1995). Similar patterns have been seen elsewhere in the world (e.g., Ekesi et al. 2009), often with dominance by *B. dorsalis* (Duyck, David, et al. 2004) or *B. tryoni* (Dominiak and Mapson 2017).

## Lures

### Parapheromones

Mature males of many Tephritidae will respond to specific parapheromone attractants (Sivinski and Calkins 1986), and these lures became the mainstay of most fruit fly surveillance programs worldwide (Quilici and Donner 2012). The operational significance of 3 particular lures—trimedlure, cuelure, and methyl eugenol—is such that fruit flies are typically grouped depending on which lure they respond to. Trimedlure is attractive to *Ceratitis* species including *C. capitata*, while cuelure attracts *B. tryoni*, *Z. cucurbitae*, and at least 55 other crop-damaging fruit fly species (Drew 1974). Methyl eugenol is strongly attractive to *B. dorsalis* and at least 22 other pest species (Drew 1974). A fourth group, including *Z. cucumis*, *B. jarvisi*, *B. oleae*, and the *Anastrepha* species, are not strongly attracted to any of the 3 main male lures but may be detected with generic attractants, typically food lures (Martinez et al. 2007).

Researchers continue to investigate new parapheromone lures with greater surveillance potential, such as ceralure (Jang et al. 2010), raspberry ketone trifluoroacetate (Siderhurst et al. 2016), zingerone (Wee et al. 2018), anisyl acetone (Royer et al. 2020), and α-copaene (Shelly et al. 2023). In some cases, more effective alternatives are not adopted because of their higher production costs (e.g., Jang et al. 2010) or the need for additional traps in existing networks. For example, in Queensland zingerone is more effective than cuelure for *B. jarvisi* (Fay 2012, Royer et al. 2017) but is less effective than cuelure for *B. neohumeralis* and *B. tryoni* (Fay 2012). Deploying both lures would substantially increase overall costs, but zingerone traps might be used specifically in mango orchards where *B. jarvisi* thrives (Dominiak and Worsley 2017).

Traps individually baited with trimedlure, cuelure, and methyl eugenol characterize fruit fly surveillance networks in NZ (MacLellan et al. 2021), Australia (Dominiak et al. 2003), California (Gilbert et al. 2013), and elsewhere (Quilici and Donner 2012) (Table 1). Lure dispensers are a key component of these traps, with a choice of wicks, plugs, and wafers. In Australia, cotton wicks are usually used as dispensers to carry both toxicants and male lures (Dominiak et al. 2011, Anderson et al. 2017). New Zealand recently changed from plugs to wafers in response to a study that found that wafers emit higher concentrations of attractants, last longer, and may catch more fruit flies than plug dispensers (Suckling et al. 2008). Improvements to polymers used in plugs and wafers continue apace (e.g., Kuzmich et al. 2021).

**Table 1.** Summary of trap architecture, lure and toxicant combinations primarily used by different states for early detection of exotic fruit flies

Country/state	Trap design	Lure	Toxicant/fly retention
New Zealand	Lynfield (plastic pottle)	Trimedlure Cuelure Methyl eugenol	DDVP strip
Australia (general use)	Lynfield/Cera (plastic pottle)	Trimedlure Cuelure Methyl eugenol	Malathion or DDVP on dental wicks
Australia (domestic assurance)	Cone traps	Trimedlure Cuelure	Alpha cypermethrin wiped inside lid of trap
California	Jackson (delta)	Trimedlure	Sticky base
	Jackson (delta)	Cuelure Methyl eugenol	Dibrom
	Multilure (plastic jar)	Torula yeast or BioLure	Drowning
	McPhail (glass jar)	Torula yeast	Drowning
	Pherocon AM (yellow sticky panel)	Ammonium acetate and protein hydrolysate	Sticky mat
	ChamP (yellow sticky panel)	Ammonium bicarbonate	Sticky panel
Hawaii	Bucket (Highland Plastics)	Trimedlure Cuelure Methyl eugenol	DDVP strip
	Multilure (plastic jar)	Torula yeast	Drowning

Appropriate baiting intervals are dependent on local climatic conditions. For example, the conversion of cuelure to raspberry ketone (the active attractant) is accelerated in moist conditions (Metcalf 1990) and possibly retarded in dry inland Australia. Similarly, baiting intervals in California are varied to partially account for climate (Gilbert et al. 2013). New Zealand refreshes cuelure and methyl eugenol lures every 12 weeks, and trimedlure every 8 weeks. Australian researchers found that cuelure can be loaded at a higher concentration to achieve a longer replacement interval. Cuelure rates of up to 9 times the current standard (2 mL) were tested in male annihilation blocks without any repellent effect (Dominiak et al. 2009). In Queensland, the standard 2 ml of male attractant is used (Fay 2012), but NSW traps are baited with 4.4 ml of cuelure and are refreshed every 6 months (Dominiak and Nicol 2010). Wicks were made up at least 1 month before they were required in the field to allow cuelure to begin to break down to raspberry ketone, which is the attractive compound, and avoid a lag in attractivity.

### Food-Based Lures

Many fruit flies do not respond to the current male lures, including the *Anastrepha* fruit flies of the Americas. In addition, adults of responsive species that have fed on particular host fruits such as tropical almond may be less attracted to the standard lures than expected (Manoukis et al. 2018). Where such species are of concern, food baits may be used, and many detection programs pair parapheromone lure traps with food bait traps (Shelly et al. 2014). These are not species-specific or gender-specific, allowing the possible detection of incursion by species that may not be under specific surveillance, or for which there is no parapheromone lure available (Martinez et al. 2007, Epsky et al. 2011). Also, food-baited traps can detect sexually immature males and adult females who would not be attracted to parapheromone lures (Henneken et al. 2022).

Food-based lures are diverse. The principal ones used for tephritids are protein-based attractants such as torula yeast or NuLure (Epsky et al. 1993) and synthetic volatile blends such as BioLure containing 2 or 3 of the components: ammonium acetate, triethyl amine, and putrescine (Heath et al. 1995, 1997). California utilizes 4 different food baits in specific trap types: torula yeast in glass McPhail traps; ammonium carbonate in ChamP traps; Pherocon AM (yellow sticky panel) traps impregnated with ammonium acetate and protein hydrolysate; and Multilure traps baited with BioLure (Quilici and Donner 2012, Gilbert et al. 2013). The mechanisms of attraction and their dependence on insect physiological state, environment, and other factors remain difficult to elucidate (Piñero et al. 2020).

While most of our discussion concerns the use of food-based lures for trapping, experience in Hawaii shows the importance of protein baits for control measures. There, GF-120 was a new commercial formulation of proteins with higher attraction than Nu-Lure, and was heavily used together with male annihilation (cuelure and/or methyl eugenol) and field sanitation. In combination with biological control, SIT, and other measures, protein was important to effectively control fruit fly species of economic importance in Hawaii (Vargas et al. 2001, Prokopy et al. 2003, Stark et al. 2004).

Relative to parapheromone lures, protein-baited traps are considered to have a more limited attraction to fruit flies, but this may vary by species. For example, in California, where similar numbers of trimedlure and protein baited traps are deployed (Quilici and Donner 2012), first detections of *C. capitata* occurred approximately as frequently in protein traps as in trimedlure traps (K. Hoffmann, pers. comm. 2012). In contrast, Dominiak and Nicol (2010) found that although protein-baited McPhail traps will catch both male and female *B. tryoni*, they were considerably less effective (about one-seventh) for males than cuelure in Lynfield traps. It seems that protein lures may be effective as in-canopy lures but may fail to attract flies from adjacent trees. For melon fly, Shelly and Manoukis (2018), working in Hawaii, found only 3.6% of released flies were recaptured from a distance of 10 m in a food-baited (torula yeast) Multilure trap. Other studies found better results, such as an effective sampling range of 28 m for *C. capitata* in a mango orchard (Epsky et al. 2010), and 30 m for *Anastrepha suspensa* in guava (Kendra et al. 2010).

A second issue with liquid protein baits is that they are short-lived and labor-intensive (IAEA 2003, Dominiak 2006). Protein-baited traps may need replenishing twice weekly and require longer to service than parapheromone-based traps (Dominiak and Nicol 2010). In addition, fly samples can degrade in the liquid protein. More user-friendly protein gels are now available (Bain and Dominiak 2022). In southern Australia, the Biotrap (Biotrap 2023) with a protein gel lure performed as well as cuelure-baited Lynfield traps for catching *B. tryoni* (Bain and Dominiak 2022), which was unexpected. New Zealand is also evaluating the protein gel-based Biotrap (Voice and MacLellan 2020).

Protein-based traps may still suffer from excess bycatch, for example, of blow flies, particularly in pastoral areas (Dominiak et al. 2003). The issues of low efficacy, frequent servicing, and excess bycatch currently preclude protein-baited traps from general use in NZ and Australia. However, these traps may still have value for trapping in the highest-risk urban areas and when no specific attractants exist for a given target species (Lasa et al. 2015).

Often, synthetic food-based lures such as BioLure are superior to torula-yeast protein lures (Gazit et al. 1998, Epsky et al. 1999, Katsoyannos et al. 1999, Papadopoulos et al. 2001) except for *Bactrocera* flies (Leblanc et al. 2010). For *C. capitata* in Australia, BioLure in Tephri or McPhail traps were superior to orange ammonia and liquid protein hydrolysate regardless of climate, tree type, or population level (Broughton and De Lima 2002). BioLure traps may catch more *C. capitata* than those baited with the parapheromone trimedlure (Epsky et al. 1999, Katsoyannos et al. 1999), depending on trap architecture (Broughton and Rahman 2017). The effectiveness of this lure is sufficient for it to be successfully used for mass trapping in Spain and Israel (Cohen and Yuval 2000, Navarro-Llopis et al. 2008). The problem of non-target captures (“bycatch”) inherent to food lures still exists with BioLure but was reduced compared with traditional protein and can be minimized further by placement strategies (Mangan and Thomas 2014).

Fruit extract lures have been used in several situations. Orange juice lure was investigated in Australia (Dominiak and Nicol 2010) but was not adopted for broad-scale use. Orange juice or hydrolyzed protein can be used for a short time to help identify the epicenter of an outbreak (Bateman 1991). Grape juice lures were used for *Anastrepha* monitoring in Latin America (Robacker et al. 2011, Epsky et al. 2015, Herrera et al. 2016). These lures have the same disadvantages as other liquid-based lures and suffer from a low attraction range.

### Combined Lures

Each of the 3 main fruit fly lures is currently delivered in separate traps spaced at least 3 m apart at any location, since early work suggested that combinations of the lures may depress trap efficacy (Hill 1986, R. Cunningham, pers. comm. cited by Cowley and Frampton 1989). However, trials in Hawaii with a combined triple lure containing trimedlure, methyl eugenol, and raspberry ketone (closely related to cuelure) and the toxicant DDVP found no reduction in efficacy compared to single-lure traps for *C. capitata*, *B. dorsalis*, and *Z. cucurbitae* (Shelly et al. 2012, Vargas, Souder, et al. 2012). Stringer et al. (2019) found similar results, except that the catch of *B. dorsalis* was significantly reduced in traps baited with a combination of trimedlure, cuelure, and methyl eugenol compared to traps baited with methyl eugenol only. Shelly et al. (2016) concluded that the combination lure may be less effective than current practice for some species. Cuelure and trimedlure can be combined without loss of efficacy for key target species, but cuelure and methyl eugenol together may experience depressed catch (Royer and Mayer 2018). In NSW, the combination of cuelure and methyl eugenol had merits in the drier inland climate but was of debatable merit in coastal Sydney (Dominiak et al. 2011). There is a need for further trials with certain lure combinations to confirm their suitability for early detection programs. Another possibility is to combine a food lure or its components with a parapheromone. Trials in Hawaii showed increased suppression of *C. capitata* when both trimedlure and BioLure were used (Vargas et al. 2018).

One of the reasons for varying results between tests of combination lures may be the relative abundance of each of the target species. In parts of Hawaii, any trap baited with methyl eugenol quickly becomes overwhelmed with *B. dorsalis* males when this species is abundant. For a delta trap, this might mean other species such as *Z. cucurbitae* land on a thick layer of *B. dorsalis*, making it easy for them to fall out of the trap or escape. In addition, a large number of *B. dorsalis* might cause behavioral interference limiting the catch of other species (Manoukis et al. 2023).

## Toxicants

Toxicants are used to prevent insect egress from dry, non-sticky tephritid traps. Significant differences between the toxicants used by different countries reflect different states of chemical registration and restriction, and different trap architectures used. Nevertheless, some combinations have become de facto international standards, such as cuelure-baited Lynfield traps with malathion or dichlorvos (DDVP) used in Australia and NZ (Dominiak et al. 2003). Malathion is stable and is active for up to 6 months (Dominiak and Nicol 2012). However, Queensland uses malathion 500 g/L (Lloyd et al. 2010, Fay 2012) while NSW and Western Australia use 1,140 g/L (Dominiak et al. 2003). The trapping program in the Torres Strait uses malathion (Huxham 2004).

Generally, toxicants are not required for traps utilizing a sticky surface for the retention of smaller fruit flies such as *C. capitata*. However, a toxicant is important to improve trap efficacy for larger species such as *Z. cucurbitae* and even *B. dorsalis* (Vargas et al. 2009, Manoukis et al. 2023). California uses dibrom (“Naled,” dimethyl 1,2-dibromo-2,2-dichloroethyl- phosphate) in Jackson traps that target *Bactrocera* and *Zeugodacus* flies because these large species may be strong enough to escape from a sticky panel. In California, the move from malathion to dibrom was dictated by the public’s reaction to an aerial application incident involving malathion, even though it is probably less toxic to mammals than dibrom (Haberman 2014).

Dibrom is not registered for any use in Australia or NZ. Instead, NZ utilizes another organophosphate, dichlorvos (DDVP). This was initially thought to have a repellent effect on fruit flies because traps using DDVP in Hawaii caught fewer flies than other traps (Vargas et al. 2003, Shelly et al. 2016). Alpha-cypermethrin performed equally well as DDVP strips and may replace DDVP in NZ (Voice and MacLellan 2020). Manoukis (2016) found that fresh “hot” DDVP may kill some flies before they even enter a Jackson trap, mimicking repellency, but suggested the effect would likely be insignificant. Effects of vapor-borne toxicants like DDVP will depend partly on trap architecture, so toxicant trials should ideally use trap and lure configurations that match those in the country of intended use. DDVP was subsequently found no more repellent in Lynfield traps than alternative toxicants bifenthrin and alpha-cypermethrin (Voice and MacLellan 2020). Surveillance trapping in Hawaii employs DDVP as a killing agent (Leblanc et al. 2012, 2014).

Another possible toxicant for use in fruit fly traps is spinetoram/spinosad (Reynolds et al. 2017). This reduced-risk insecticide was applied successfully against tephritids in Hawaii and other areas decades ago (Peck and McQuate 2000) and is used today as part of male annihilation in California and elsewhere (Vargas et al. 2014). Spinosad’s relatively high rate of photodegradation (Tomkins et al. 1999) makes it suitable for bait sprays, but may be problematic in extended-use traps with clear sides.

Health and safety considerations partly dictate which toxicants can be used in each country. The number of pesticides for fruit fly activities continues to decline and surveillance managers should not rely on any one toxicant (Dominiak and Ekman 2013). Ideally, whatever toxicants are used should be purchased pre-packaged to minimize handling hazards. Until 2017, NSW authorities were manufacturing their own wick/lure/toxicant combinations; they now purchase the entire trap unit, including lures and toxicants, pre-built (Biotrap 2023).

## Trap Architecture

California uses Jackson traps as the main trap design. These comprise a delta trap with a sticky mat to collect insects (IAEA 2003). However, once the mat has accumulated one layer of insects, subsequent insects are not retained and populations will be underestimated. This is only a potential problem in high pest populations or when dust and similar debris fouls the sticky mat. To supplement these, California utilizes glass McPhail traps, ChamP traps, and Pherocon AM (yellow sticky panel) traps impregnated with ammonium acetate and protein hydrolysate, and Multilure traps baited with BioLure (IAEA 2003, Quilici and Donner 2012, Gilbert et al. 2013). The heavy glass McPhail traps are favored in California for their stability during the region’s strong offshore wind events (J. Leathers, pers. comm.) but are not commercially available so the state has been transitioning to plastic Multilure traps. Monitoring in Hawaii, the other US state we focus on, has employed bucket traps for male lures and multilure traps for wet protein lure (torula yeast) (Leblanc et al 2014).

Fruit fly surveillance in the Torres Strait between Australia and Papua New Guinea uses Paton traps at permanent trapping sites. The lighter Steiner trap is still used when additional trapping is required (Huxham 2004). Fruit fly trapping in Papua New Guinea and the tropical north of Australia uses modified Steiner traps (Iamba et al. 2021). However, Jackson sticky traps were found to be twice as effective as the standard Steiner traps in Victoria, so Jackson traps became the standard for a decade in southeastern Australia (O’Loughlin et al. 1983). Subsequently, Lynfield traps were found to be more effective than Jackson traps for *B. tryoni* (Cowley et al. 1990). Lynfield traps consist of a 1 L cylindrical clear plastic pottle (120 mm in depth and diameter), a lid, and a lure dispenser (Cowley et al. 1990, Dominiak and Nicol 2010). Four 25-mm holes are drilled at equally spaced locations around the sides of the pottle to allow the lure vapor to exit the trap and for insects to enter. An additional four 2-mm holes are drilled in the bottom for water drainage. Lure dispensers comprising cotton wicks—4 dental cotton rolls (each 10 mm × 40 mm long) held together by a wire clamp—are suspended from the middle of the Lynfield trap lid. The wick hangs at about the same level as the ingress holes in the side wall of the trap. One advantage of the Lynfield traps is their large capacity which makes them a better option in high fly populations, compared to Jackson traps. In addition, flies are loose and do not have to be removed from sticky mats.

Recently, NZ and Australia compared Lynfield traps with Biotrap and cone traps (Dominiak et al. 2019, Voice and MacLellan 2020, Bain and Dominiak 2022). The Biotrap was developed in Australia and has some design commonality with MacPhail traps. Biotrap traps are popular in some regions (Bain and Dominiak 2022). Cone traps were developed in Spain for *C. capitata* surveillance. They have a clear lid and yellow sides, exploiting the finding that various fruit fly species are attracted to yellow (Hill and Hooper 1984, Katsoyannos 1987). One problem with Lynfield traps is that trapped flies are drawn to the clear sides and may stumble out of the entrance holes before they die or contact the toxicant. In cone traps, the ingress holes have invaginations in the yellow wall, with a tunnel of about 1 cm helping to prevent accidental escape (Dominiak et al. 2019, Voice and MacLellan 2020). In addition, the clear lid draws trapped flies away from the entrance holes and up to the toxicant, which may be painted on or suspended from the lid. Dead flies fall to the bottom of the cone, where a clip-on trapdoor allows inspectors to efficiently collect them, even in windy conditions when specimens may blow out of an open Lynfield trap during sample collection. Administratively, cone traps are transported flat-packed with lids, potentially saving costs in distributing traps to surveillance areas. Lynfield bases do not flat pack and require considerable space for storage or transport (Dominiak et al. 2019).

A wide range of modern commercial fruit fly traps have been designed for mass trapping, most of which might be considered variants of the Lynfield or earlier traps. Several studies have compared their performance (e.g., Lasa et al. 2014, Broughton and Rahman 2017, Dominiak et al. 2019, Bain and Dominiak 2022) and found that most performed well under different conditions and it is unlikely that any trap one trap will suit all circumstances. Therefore, the adoption of any particular trap design may be determined more by cost and convenience than by their relatively small differences in efficacy.

## Seasonal Trap Deployment

**Fig. 3.**
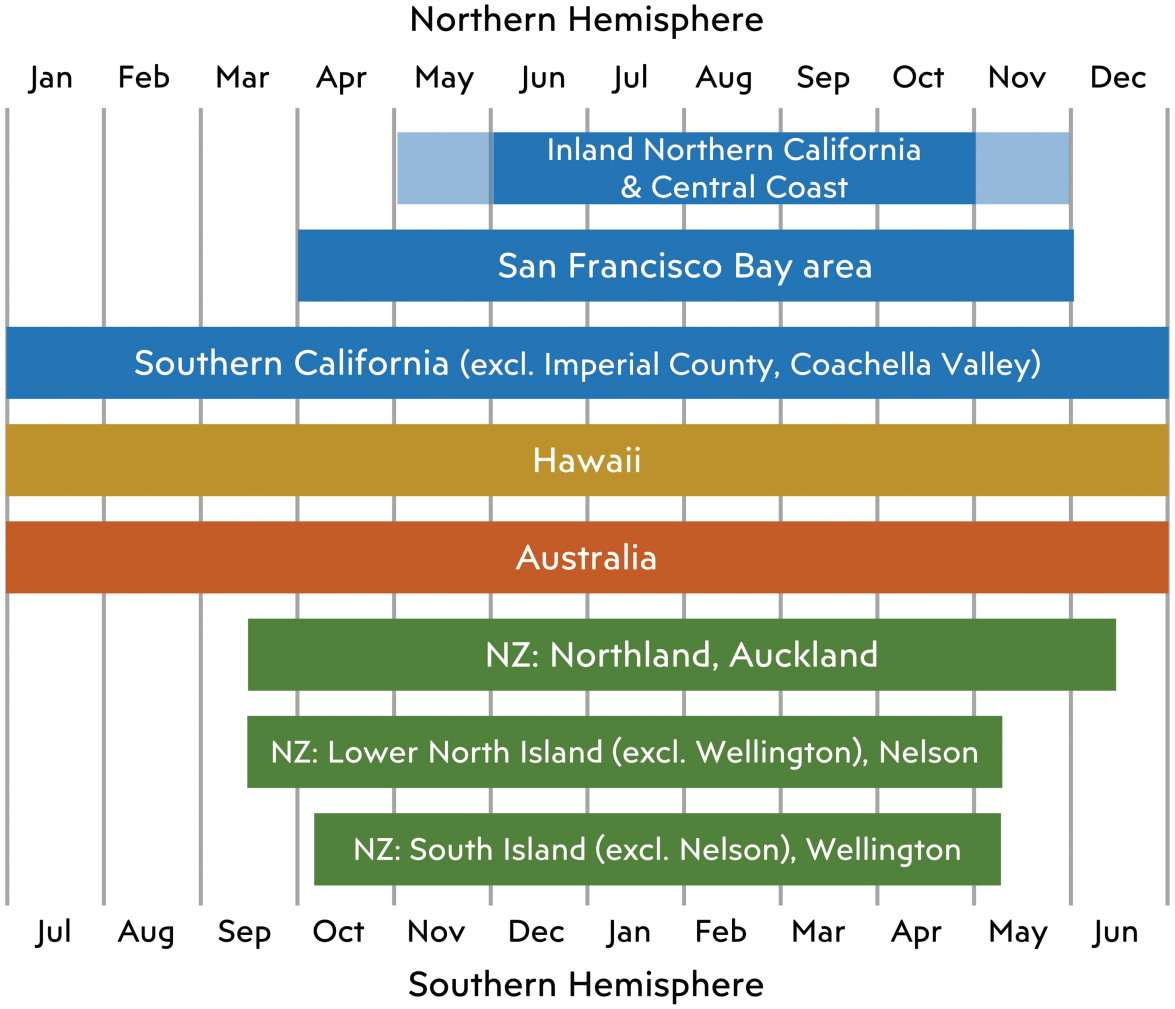
Comparison of fruit fly detection trapping seasons in different regions, from midwinter to midwinter.

States differ in the portion of the year they deploy fruit fly surveillance traps for detection of new incursions (Fig. 3). In Australia, the Code of Practice for the Management of Queensland Fruit Fly (Department of Primary Industries 1996) specifies year-round trapping, based largely on the risk of fruit flies to be spread domestically at any time of year. Similarly, trapping is conducted year-round in southern California, except in Imperial County and Coachella Valley in the height of summer (Gilbert et al. 2013). Further north in California, winter cold limits fruit fly persistence (De Villiers et al. 2016, Szyniszewska et al. 2020). In the San Francisco Bay area, trimedlure, methyl eugenol, and torula yeast traps are deployed from April to November, while cuelure traps are set out from June through October. These periods are shortened by a month on either end in other urban areas of northern California (Gilbert et al. 2013). Traps are deployed for 6 months in inland Northern California and the Central Coast, but the exact timing varies to allow counties to take advantage of local knowledge on the availability of host fruit when placing traps.

In NZ, the fruit fly surveillance season has been adjusted several times in response to new knowledge and new technologies. Initially, traps were deployed year-round in the northern North Island and elsewhere removed during winter (Somerfield 1989). With the introduction of Lynfield traps, all locations north of Christchurch were trapped year-round and more southerly locations from September through April (Cowley et al. 1990). In 1999, all winter trapping was stopped when it was realized that it contributed little to fruit fly surveillance. More recently, Kean and Stringer (2019) modeled the effects of seasonal temperatures on trap catches of *C. capitata*, *B. tryoni*, and *B. dorsalis* in their native and invaded ranges, and used this to determine the optimal trapping periods for NZ locations. Similar results were obtained by considering the proportion of the days that air temperatures are above the threshold for male flight (Kean 2016). In response, the dates for starting and ending surveillance were adjusted and fruit flies are now trapped from mid-September to mid-June in the north and shortened by about a month on either end in the south (Fig. 3; MacLellan et al. 2021).

Hawaii has a mild tropical climate with little seasonal variability, so trapping efforts for surveillance generally need to be conducted year-round. Recent efforts have focused on year-round trapping in areas around ports of entry on the island of Oahu (Leblanc et al 2014) but in the past, there was island-wide trapping on Oahu year-round (Leblanc et al 2012).

## Relative Trapping Effort

Trap spacing practices vary considerably across the reviewed states (Table 2). Most states trap predominantly in urban areas, which are considered to have elevated risk of entry and establishment due to human-vectored dispersal (Liebhold et al. 2006, Dominiak and Coombes 2009) and the availability of poorly managed backyard fruit trees. The details of many countries’ exotic fruit fly trapping networks were reviewed by Quilici and Donner (2012), so here we briefly summarize and update their results.

**Table 2.** Standard trap grid spacings used for early detection of exotic fruit flies. Data updated from [Quilici & Donner (2012)]

Country/state	Lure type	Area	Nominal trap spacing
New Zealand	Trimedlure, cuelure	High risk (urban)	400 m
	Methyl eugenol	High risk (urban)	1,200 m
Australia: general	Trimedlure, cuelure, methyl eugenol	Urban	400 m
		Production areas	1,000 m
Australia: New South Wales	Trimedlure, cuelure, methyl eugenol	First ports of entry	5,000 m
California	Various	Urban	725 m

**Fig. 4.**
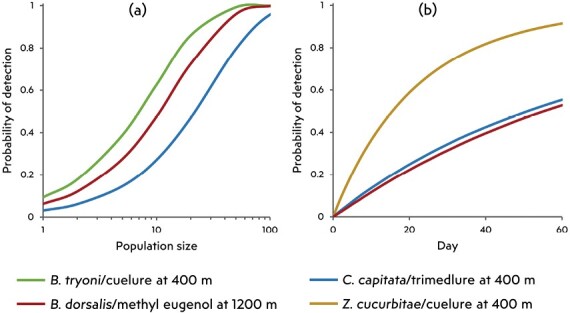
Cumulative probability of detection for different species/lure combinations at 2 different trap spacings. a) Probability of detection with population size (Kean, 2017). b) Mean detection over time from 250 simulations, each of 200 trappable flies, from the TrapGrid model (Manoukis et al. 2014).

New Zealand deploys around 3,500 cuelure-baited traps each year, the same number of trimedlure traps, and about 800 methyl eugenol traps (MacLellan et al. 2021). Cuelure and trimedlure traps are spaced at approximately 400-m intervals in a grid across areas with relatively high identified risk. Methyl eugenol traps are placed more sparsely at 1,200 m intervals, reflecting their higher attraction radius. Kean (2017) estimated that these densities would give a high probability of detecting incipient populations before they reach 40–100 adult males (Fig. 4a), and such populations may be successfully eradicated (Suckling et al. 2016). Across NZ as a whole, and considering the estimated risks outside trapped areas, the current trapping program was estimated to give a 59% probability of detecting at least one of the first 100 *C. capitata* males present. The equivalent estimates for *B. tryoni* and *B. dorsalis* were 83% and 66%, respectively. By the time a new population of any of these species had produced 10,000 males, there was estimated to be a > 99% chance of detection by trapping or passive surveillance (Kean 2017). Similarly, simulation models such as “TrapGrid” (Manoukis et al. 2014) can extrapolate from the decline in trap captures with distance (e.g., Manoukis et al. 2015, Manoukis and Gayle 2016) to estimate the temporal cumulative probability of detecting fruit fly populations in trapping grids with particular configurations (Fig. 4b; Fang et al. 2022).

Over all its states and territories, Australia deploys around 4,800 traps (equal numbers of trimedlure, cuelure, and methyl eugenol traps) around ports of entry. Cuelure and methyl eugenol traps are deployed across multiple sites in the Torres Straits between Cape York and Papua New Guinea (Quilici and Donner 2012). The individual states of Australia deploy an additional c. 25,000 fruit fly traps, usually at 400-m intervals in urban areas and 1,000 m in rural areas (Table 2). For example, South Australia runs approximately 7,500 traps (a similar number as used in NZ) but added more than 18,000 traps during recent eradication efforts against *B. tryoni* and *C. capitata* (Department of Primary Industries and Regions 2021). Meanwhile, Tasmania declares freedom from fruit flies based on approximately 1,000 traps deployed annually in urban areas (Blake 2019).

Recently, NSW adopted a much sparser trapping network than was previously used, with traps deployed no closer than 5 km apart everywhere, including in urban areas. No exotic fruit flies have been detected in NSW in the last 20 years (Dominiak 2020) and risk management practices have improved markedly (e.g., Dominiak 2019, van Klinken et al. 2020). Current thinking is that fruit fly incursions into NSW are most likely to be linked to travelers moving small quantities of fruit, with a resultant low chance of establishment (Maelzer 1990, Dominiak and Coombes 2009). Furthermore, if *C. capitata* were to enter from Western Australia, it would likely be prevented from establishing by the entrenched endemic population of *B. tryoni* (Dominiak and Mapson 2017). These arguments gave the NSW authorities the confidence to markedly reduce their surveillance efforts for exotic fruit flies.

California employs at least 3 times as many fruit fly traps as Australia and NZ combined, reflecting the relatively high rates of entry and establishment there. Arrays of approximately 25,000 trimedlure, 20,000 cuelure, 20,000 methyl eugenol, and 27,500 food-based traps are deployed across urban areas, together with around 700 sticky panels for detecting *Rhagoletis* fruit flies (Quilici and Donner 2012). Similar numbers are used in Florida, while Texas targets *Anastrepha* species from Central America using food-based lures (Quilici and Donner 2012). In Hawaii, trapping with male lures was a key method to measure the effectiveness of the area-wide IPM program (Vargas et al. 2008). A 20-fold reduction of *Z. cucurbitae* catch in Waimea was a leading indicator of successful control measures. Similar results were seen elsewhere for a total of 653 farms state-wide (Vargas et al. 2008).

**Fig. 5.**
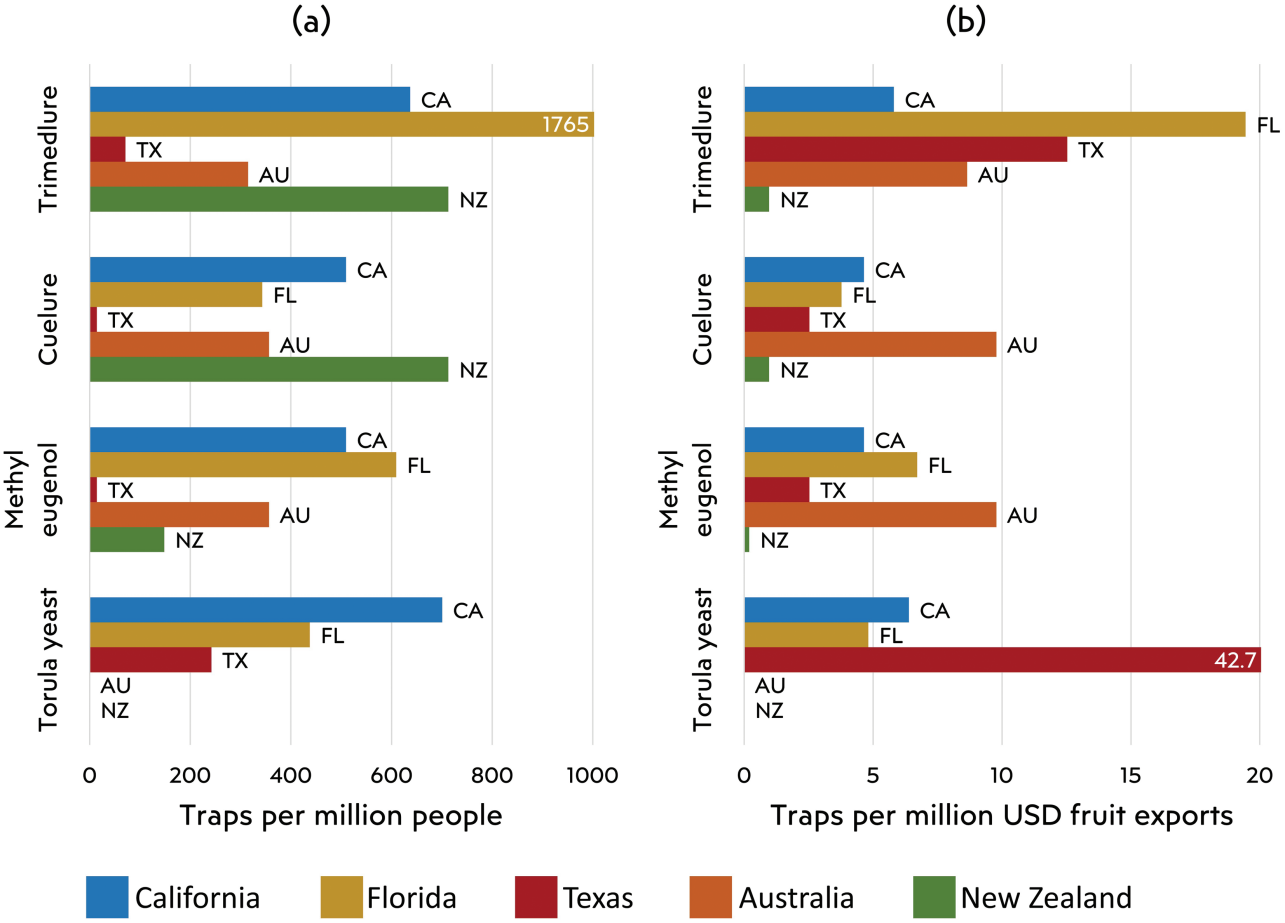
Comparison of the number of fruit fly surveillance traps deployed in 2 different states, relative to a) human population size and b) value of fresh fruit exports. Data are updated from [Quilici & Donner (2012)] and national statistics authorities.

To compare trapping effort across states, it is useful to contextualize these numbers by risks and benefits. If propagule pressure is determined largely by human activities (Maelzer 1990, Dominiak and Coombes 2009), then traps per million people may indicate how different states perceive fruit fly propagule pressure (Fig. 5a). In these terms, NZ’s cuelure trapping is high relative to other states, but may appropriately reflect the importance of detecting and excluding *B. tryoni* and other cuelure-responsive threats. Trimedlure trapping is similar between NZ and California but much lower than Florida. New Zealand’s methyl eugenol trapping is low compared to Australia, California, and Florida, where these traps are deployed at the same density as other lure types.

Another way to compare trapping effort is to consider the resource being protected. Through this lens, NZ’s trapping effort relative to the value of fruit exports is low (Fig. 5b). Florida and Texas invest relatively more in trimedlure traps, perhaps indicating elevated concern about *Ceratitis* species, or possibly to compensate for the relatively short attraction radius of this lure (Manoukis et al. 2015). Texas’s local threat of *Anastrepha* influx from Mexico is reflected in its relatively high investment in torula yeast traps. And Australia invests relatively heavily in cuelure and methyl eugenol in response to the local threats from *Bactrocera* species, particularly from Asia (Fig. 5b).

## Local Trap Placement

New Zealand and California use host preference lists to prioritize trees in which to hang fruit fly traps (Gilbert et al. 2013), though in urban areas the choices can be limited. The plant species on which traps are suspended is of key importance in the early detection of *C. capitata* (Papadopoulos et al. 2001). We speculate that this is because the relatively short attraction radius of trimedlure (Manoukis et al. 2015) and short dispersal flight distance of *C. capitata* (Dominiak 2012) contribute to traps having greater efficacy when placed in host trees with active *C. capitata* populations. A review of the ranked host list for *C. capitata* is now available (Dominiak and Taylor-Hukins 2022) to better inform trap placement.

In NSW, Mo et al. (2014) found that *B. tryoni* were most likely to be trapped in pome trees; apples are not a primary host but can readily be infested (Follett et al. 2021). Follett et al. (2021) created a host suitability index to rank the capacity of different hosts to support the fruit fly life cycle. Some hosts, such as guava (*Psidium guajava* L.), are known to be more suitable than others (Woods et al. 2005, Lloyd et al. 2010). Traps placed in these preferred hosts may be more likely to provide an early warning for increasing populations. A full review of ranked hosts for *B. tryoni* and *B. dorsalis* is yet to be published.

Trees with fruit will slow down the movements between trees (Hendrichs and Hendrichs 1990, Dalby-Ball and Meats 2000) so the consensus is that traps should be placed in fruiting trees if available. De Lima et al. (2011) found benefits for detection in moving *B. tryoni* traps throughout the season to keep them in trees with mature fruit. In NZ, inspectors move fruit fly traps to trees with the most ripening fruit on the same property (MacLellan et al. 2021). Generally, fruit flies may be more likely to be found in urban areas rather than forests or orchards because the diversity of fruit trees provides a higher likelihood that host fruit is available at a given time (Raghu et al. 2000).

Almost universally, traps are hung at about 1.5 m above the ground (Dominiak et al. 2003, Royer et al. 2020, Iamba et al. 2021, MacLellan et al. 2021) as this is a convenient height for trap inspectors and often the widest part of a fruit tree canopy. Within a site, traps are placed at least 3 m apart to avoid the potential for interference. Trap sites in Australia typically contain 3 traps, each in a different tree (Gillespie 2003, Dominiak 2020) and a different lure in each trap. Host trees must be about 4 m apart to minimize interference between lure plumes (Hill 1986). Similar protocols are followed in NZ (MacLellan et al. 2021) and California (Gilbert et al. 2013).

## Trap Inspections and Servicing

Generally, trap inspections everywhere are conducted fortnightly except in sensitive states under high propagule pressure or in an emergency response procedure. The Australian mainland Code of Practice specifies weekly inspections except during the winter months of June to October when fortnightly trap inspections are used (Bateman 1991, Dominiak et al. 2003). Some food-based lures may degrade rapidly and require weekly servicing (Gilbert et al. 2013).

New Zealand’s wafer lures are replaced every 12 weeks for cuelure and methyl eugenol, or 8 weeks for trimedlure. The change from plug dispensers to wafers was informed by locally conducted lure degradation studies that suggested these practices were adequate (Suckling et al. 2008). Cuelure wicks are refreshed only 6 monthly in NSW because parallel work on the Male Annihilation Technique suggested that residual lure and toxicant from the initial dose of 4.4 ml of cuelure would still be above the minimum standard for effective attraction (Dominiak et al. 2011). Where DDVP toxicant is used in Australia and NZ, these strips are replaced every 2 months. In California, 2 g trimedlure gel plugs are replaced every 4 (in summer) to 12 weeks (winter), in accordance with temperature-based degradation curves (Gilbert et al. 2013).

In California, inspectors replace the sticky inserts in Jackson traps monthly, or more often as needed (Gilbert et al. 2013). Suspected exotic specimens are not removed from sticky surfaces but are submitted as-is to a diagnostic laboratory for identification. Where traps with toxicants are used, inspectors remove the individual dead insects for identification. For example, NZ inspectors submit all fly-like specimens within a size range that encompasses the fruit flies of concern (MacLellan et al. 2021). Australia, NZ, and California all use audit flies to seed traps and test the entire trap retention, detection, and reporting system. Audit intervals vary across countries.

## Data Capture, Analysis, and Review

A range of integrated technologies are used to record and map trap locations, capture digital trap records, track specimens, audit and summarize data, and manage notifications (Schellhorn and Jones 2021). For example, the California Department of Food and Agriculture created a data collection system, CalTrap, that is customized specifically for the state’s requirements (California Department of Food and Agriculture 2021). In Australia, Victoria developed Trapbase, a database built from SharePoint lists and a mobile application, enabling automatic reporting for the Commonwealth government. New South Wales has recently adopted Trapbase, dropping their own bespoke PestMon digital system (Dominiak et al. 2007). South Australia and Western Australia have also transitioned to Trapbase, and other states are evaluating the system. New Zealand’s fruit fly trapping data are digitally collected and curated by an operational contractor.

It is likely that automated trapping, meaning remote detection and/or identification of a catch (Potamitis et al. 2017), will soon be operationalized for fruit fly surveillance. In November 2022, NZ deployed 60 RapidAIM automated traps targeting *B. tryoni* (Ministry for Primary Industries 2022). This particular trap uses a capacitance sensor to identify any insect entering the trap, but other solutions may employ optical imagery, wing-beat frequency, or the amount of electric current required to surround and kill the insect (Schellhorn and Jones 2021). Generally, these automated systems also deliver real-time reporting, and in areas where target fruit flies are rare or absent this may alleviate the need for manual trap inspections.

This is a rapidly developing area, and such tools will continue to decline in price and improve in accuracy. However, widespread operational use will take time, as any significant change from current practice would need approval by international trade partners, a process that can take several years. Meanwhile, such technologies might be incorporated into domestic trade, perhaps as part of a systems approach (Dominiak 2019, van Klinken et al. 2020).

## Discussion

Our review highlights the diversity of approaches to exotic fruit fly surveillance trapping currently conducted across 4 Pacific states. The choice of trap, lure, dispenser, toxicant, and bait concentration may be partly dictated by local factors such as the target species, available (registered) chemicals, and climate. These choices help determine the efficacy of traps and the optimal trap deployment in space and time, though experiments and modeling are only recently starting to address this in a systematic way. Currently, most states follow similar practices around trap inspection, servicing, and data handling, but these processes are likely to be disrupted by emerging automated trap technologies. Ultimately, different practices can be traced back to the unique fruit fly risk profiles faced by each state, particularly the suite of fruit flies already present and those that threaten from nearby.

States which are free from economically damaging fruit flies, such as NZ, South Australia, and Tasmania, have an important advantage in being able to use specific parapheromone lures to minimize bycatch and facilitate rapid diagnosis of trapped specimens. In contrast, fruit fly endemic areas such as eastern Australia and Hawaii trap considerable volumes of bycatch, including non-economic fruit flies. For instance, NSW has many endemic non-economic tephritids, adding to bycatch and identification service costs (Dominiak 2020). In Hawaii, methyl eugenol surveillance traps would be rapidly overwhelmed by local *B. dorsalis*, and cuelure baited traps by *Z. cucurbitae* in many areas. Where abundant bycatch may saturate traps, this can influence the choice of trap architecture and dictate servicing intervals.

The situation in Hawaii is complicated not only by the limited usability of parapheromone lures for surveillance trapping due to large standing populations of pestiferous tephritids that tend to overwhelm traps but also by its geographic configuration. The most recent surveillance effort in the state has run since 2006, and since 2009 this has focused on ports of entry on the island of Oahu (Leblanc et al 2012, 2014). This is justified by Oahu being the most heavily populated of the Hawaiian islands (with > 70% of the state’s residents), and receiving the bulk of domestic and international flights. However, passengers to Oahu often transit to other islands with more agricultural land. New invasive fruit flies might therefore not necessarily be detected outside the international airport in Honolulu (Oahu), and the impact of an establishment might be greater on other islands.

The most recent surveillance effort led by USDA-APHIS on Oahu (2006–2023) was extensive for that island. Approximately 350 sites were sampled about 17,500 times per year from 2009 to 2013, yielding a total capture of 8.5 million flies from the 4 established species (olive fruit fly was not established during that time). Over the whole period starting in 2006 a single exotic fruit fly, a *Bactrocera albistrigata* (de Meijere) male, was detected on Oahu in 2017 (T. Shelly, pers. com.). It is difficult to conclude that this is due to low propagule pressure in Hawaii, but the reasons for such a low rate of detection of exotic fruit flies in the face of large passenger volumes are difficult to divine.

Traps using food-based lures are particularly prone to non-tephritid bycatch (Leblanc et al. 2010). In addition, the lures may be difficult to handle (Dominiak 2006), though new gel-based formulations may solve many of the issues associated with liquid protein baits (Bain and Dominiak 2022). Generally, food-based lures are less attractive than parapheromones, but their use is justified in areas such as California, Texas, and Florida which face significant threats from species that do not respond to the current suite of parapheromones. Also, food-based lures may complement parapheromones by targeting adult females (Henneken et al. 2022) and in this way may even perform as well as trimedlure, the weakest of the 3 standard parapheromones, under some conditions (Epsky et al. 1999).

All reviewed states utilize the parapheromones trimedlure, cuelure, and methyl eugenol (Table 1), though only NZ varies the density of traps to reflect the relative efficacies of these lures (Table 2). A better understanding of the factors influencing the effective sampling area of fruit fly traps (e.g., Manoukis et al. 2015) would improve the interpretation of trap catches (Manoukis et al. 2014). This knowledge might allow spatial trap and lure deployment to be optimized to achieve surveillance sensitivity targets (Kean 2017). There is considerable current research to develop new parapheromones that may ultimately widen the range of species that can be effectively trapped, but much of this work is motivated by local threats (e.g., Wee et al. 2018) so it may further increase the diversity of fruit fly surveillance approaches used across states.

The reliance on particular toxicants, such as DDVP and other organophosphates, is a potential vulnerability for some tephritid trapping systems. Although a range of toxicants are used internationally, not all have been registered for use in particular countries, leaving some trapping systems without fit-for-purpose alternatives if DDVP or similar chemicals become unavailable. Some new automated trap types will not require toxicants (Schellhorn and Jones 2021), and this is just one of many ways that such technologies are likely to disrupt current fruit fly surveillance practices in the near future.

The diversity of fruit fly risks and surveillance approaches used across countries in the Pacific region makes it clear that a “one-size-fits-all” approach would not be appropriate. Every state has tailored its approach to address its own individual risks and circumstances including the species threats, propagule pressure, climate, existing fruit fly fauna, and consequences of a new exotic establishment. Current systems are not perfect, as evidenced by establishments in Hawaii and California (Fig. 2), but systems continue to evolve and improve within the time frames dictated by international trade assurances.

The main challenge for the future may be whether international trade assurances can keep pace with the accelerating need for changes in fruit fly surveillance systems resulting from new lures, climate change effects on lure degradation, automated traps, and shifting risk profiles as invasive fruit flies continue to spread internationally. Regulators need evidence-based and biologically informed surveillance strategies for fruit flies, underpinned by an understanding of the diversity of international practices.
